# A Comparative Study of Transmembrane Diffusion and Permeation of Ibuprofen across Synthetic Membranes Using Franz Diffusion Cells

**DOI:** 10.3390/pharmaceutics2020209

**Published:** 2010-05-18

**Authors:** Shiow-Fern Ng, Jennifer Rouse, Dominic Sanderson, Gillian Eccleston

**Affiliations:** 1 Faculty of Pharmacy, Universiti Kebangsaan Malaysia, Jalan Raja Muda Abdul Aziz, Kuala Lumpur, 50300, Malaysia; 2 Strathclyde Institute of Pharmacy and Biomedical Sciences, University of Strathclyde, Taylor Street, Glasgow G4 0NR, UK; 3 GlaxoSmithKline, Pharmaceutical Development, Harlow, CM19 5AW, UK

**Keywords:** Franz diffusion cells, synthetic membranes, ibuprofen drug diffusion, transdermal absorption, *in vitro* absorption

## Abstract

Synthetic membranes used in Franz diffusion cells for topical formulation quality assessment should provide least resistance to drug diffusion. In this study, the diffusion rates of ibuprofen across thirteen membranes were determined using Franz diffusion cells. Correlation of the membrane thickness, pore size and MWCO with drug fluxes was also made. The drug diffusion results showed that the porous membranes were categorized into high-flux (8–18 mg/cm^2^/h) and low-flux (0.1–3 mg/cm^2^/h) membranes. The drug fluxes did not show strong correlations (r^2^ < 0.99) with membrane parameters. Synthetic membranes can give variable drug fluxes, thus investigators should be careful in choosing membrane for formulation quality assessment.

## 1. Introduction

The use of Franz diffusion cell to assess skin permeability has evolved into a major research methodology, providing key insights into the relationships between skin, drug and formulation [1,2]. Such testing is not only highly useful in the design and development of novel formulations, but also for toxicity screening [3] and quality control [4,5,6]. Franz diffusion cells are normally used with excised human or animal skin. However, when biological skin is not readily available, synthetic membranes are employed. The synthetic membranes employed in Franz cell drug diffusion studies have two functions: simulation of the skin [7,8] and quality control [9]. Polymethylsiloxane (PDMS) is an example of a synthetic membrane that is often employed to simulate the skin because it is hydrophobic and possesses rate-limiting properties like skin [8,10]. On the other hand, synthetic membranes for quality control should have minimum diffusion resistance to drugs and only act as a support to separate the formulation from the receptor medium [4,5,6]. The synthetic membrane should be a ‘continuous’ medium of the receptor media. Such synthetic membranes often contain pores, henceforth termed ‘porous membranes’. 

In the past two decades, much research has been carried out in the assessment of topical drug diffusion using porous synthetic membranes. A wide selection of porous synthetic membranes, ranging from semisynthetic to synthetic polymers, is commercially available on the market. Usually, the porous synthetic membranes used (e.g., cellulose acetate, polysulfone) in Franz diffusion cells are ‘borrowed’ from separation and filtration applications. From the literature, investigators have employed synthetic membranes of a diverse range of materials, pore sizes and thicknesses [11,12,13,14,15,16,17,18,19,20,21,22,23,24,25,26,27]. The common membranes are silicone, cellulose and polysulfone membranes. 

The Food and Drug Administration (FDA) has suggested that simple, porous synthetic membranes are suitable for assessing topical formulation performance as they act as a support yet are not rate-limiting barriers [28]. Shah and co-workers from FDA [29] used different microporous membranes, namely pure cellulose acetate, cellulose and polysulfone of similar pore sizes and thicknesses to examine the permeation of hydrocortisone (HC) from two commercial creams. They found that the HC flux was consistent irrespective of the types of synthetic membrane [29]. On the other hand, Wu and co-workers [30] evaluated 10 types of commercial synthetic membranes such as polysulfone, cellulose mixed esters, polytetraflouroethylene and polypropylene with different pore size and thickness to evaluate the nitroglycerin drug release from commercial ointments. The study results categorize the synthetic membranes into two groups: group 1 showed higher drug permeation compared to group 2. Group 1 consisted of polysulfone, acrylic polymer, glass fiber, silicone, and mixed cellulose ester. Group 2 include PTFE-polyethylene, mixed cellulose ester (of greater thickness), polypropylene, and PTFE. However, the authors did not further elaborate on the results [30]. In another study, the effect of membrane types upon ketoprofen drug release from a gel was studied [31]. Comparison were made between two filter membranes, namely nylon (0.2 μm pore size, 129.3 μm thickness) and Celgard polypropylene (0.05 μm pore size, 26 μm thickness) and a nonporous silicone membrane (57 μm). The study noted that nylon has the least rate-limiting effects for ketoprofen even though it is a thicker membrane [31]. Different types of synthetic materials and dosage forms were tested, but the choice of synthetic membranes was not clearly explained. 

In order to have a fair evaluation of drug diffusion through each membrane, the most appropriate method would be to evaluate each membrane under standardized experimental conditions, carried out by the same operator as well as using the same drug on validated Franz cell equipment. The main aim of this study is to compare the influence of 13 different types of commercial synthetic membranes upon drug diffusion in the validated Franz cells using ibuprofen (MW 206.4, log P 3.5, pKa 4.5) as the model drug.

## 2. Experimental Section

### 2.1. Synthetic membranes

Table 1 shows the synthetic membranes employed in Franz diffusion cells experiments in this study. The membrane thickness, molecular-weight cut off (MWCO) range, nominal pore size, porosity and individual manufacturers are also shown. They are grouped as ‘polymeric’ and ‘cellulose’ based. Note that all membranes listed are porous because this study focused only on membranes for quality control purposes. PDMS (non-porous) was listed for comparison purposes. 

**Table 1:** Summary of the synthetic membrane properties. All values are nominal provided by manufacturers (ρ - membrane porosity, τ – membrane tortuosity).

Membrane	Polymer^a^	MWCO (kDa)	Pore size (µm)	Thickness(µm)	ρ (%)	τ	Source	Batch no.
*Cellulose-based*								
Visking	RC	12-14	-	20^b^	-	-	Medicell (London, UK)	DTV12000.05.000
Cuprophan	RC	10	-	10^b^	-	-	Medicell (London, UK)	N/A
Benzoylated tubing	RC	1.2-2	-	35^b^	-	-	Sigma (Dorset,UK)	074K7012
Cellulose ester	CE	0.5	-	80^b^	-	-	Spectrumlab (California)	131060
Cellulose nitrate	CN		0.45	125	66-84	-	Whatman (UK)	N/A
*Polymeric-based*								
AN 69	PAN	40	-	25^b^	-	-	Hospal (Huntingdon, UK)	N/A
Biodyne	PA	na	0.45	152	50-	-	Pall (Portsmouth, UK)	b- 50046, c-189051
Supor	PES	na	0.45	145	75	~1-1.5	Pall (Portsmouth, UK)	55083
Tuffryn	PS	na	0.45	145	80	~1-1.5	Pall (Portsmouth, UK)	60669
Nuclepore	PC	na	0.1	10	60	~1	Whatman (New Jersey, USA)	6018023
Cyclopore	PC	na	0.1	10	8	~1	Whatman (New Jersey, USA)	060.0131/6E8/L-3-L
Celgard 3500	PP	na	0.05	20	4	-	Hoechst (New Jersey, USA)	293485
Silicone	PDMS	na	-	400	35-48	-	SAMCO (Nuneaton, UK)	19T0.3-1000-60M1

^a^ RC - Regenerated cellulose, CE - Cellulose esters, CN - Cellulose nitrate, PAN - Polyacrylonitrile, PA – Polyamide (nylon), PES - Polyethersulfone, PS - Polysulfone, PC - Polycarbonate, PP - Polypropylene, PDMS – Polydimethylsiloxane. ^b^ dry thickness measured using a digital micrometer (Mitutoyo, UK).

### 2.2. Franz diffusion cells apparatus

The drug diffusion tests were undertaken on a validated tailor-made Franz cell array donated by GlaxoSmithKline (Harlow, UK). This apparatus consists of 27 tailor-made donors, receptors, and four heated magnetic stirrer chambers each containing eight blocks into which the Franz cells are placed. The effective diffusion area of the Franz cells was 59.6 ± 3.1 mm^2^ and receptor volume was 11.7 ± 0.1 mL. 

### 2.3. Other materials

Sodium hydroxide was obtained from BDH Laboratory Supplies (Poole, UK). Ibuprofen (99%) was supplied by Medex (Northants, UK). Helium gas for deaeration was supplied by BOC gases Ltd (Guilford, UK). 

### 2.4. Preparation of ibuprofen saturated solution

Ibuprofen saturated solution was prepared in the standard receptor fluid, 0.1 M sodium hydroxide, by first adding about 1.5 g of ibuprofen into 50 mL of 0.1 M sodium hydroxide. Subsequently, the suspension was agitated for at least 1 h in a shaking waterbath at 60–65 ºC. Undissolved solid was filtered at the same temperature and the solution was allowed to cool to 33 ºC. At this temperature, ibuprofen crystal growth was visible. The pH of the ibuprofen saturated solution was measured.

The ibuprofen saturated solution was prepared fresh for each Franz cell run. Mass balance was carried out to ensure the reproducibility of each ibuprofen saturated solution preparation. This was performed by comparing the mass of ibuprofen added and the ibuprofen excess filtered after every preparation. The coefficient of variation for mass balance was set to be within 5%. 

### 2.5. Ibuprofen Saturated solubility

The saturated solubility of ibuprofen in 0.1 M sodium hydroxide at temperature 25, 33, 37 and 60 ºC was determined. The ibuprofen saturated solutions were prepared as above. The ibuprofen solubility at 60 ºC was determined immediately after filtration at that temperature. For solubility determination at 25, 33 and 37 ºC, the filtered saturated solutions were each filled into a 7.5 mL vial, closed with screw cap and stored at the respective temperatures. After 24 h, the solutions were passed through 0.2 µm polysulfone filters to remove excess ibuprofen crystals. The ibuprofen solubility was determined by diluting the solution serially in 0.1 M sodium hydroxide and assaying for ibuprofen using UV spectrophotometer at wavelength 272 nm. The solubility at each temperature was determined in triplicates using fresh ibuprofen saturated solution each time.

### 2.6. Preparation of ibuprofen crystals

Ibuprofen saturated solution was prepared at 60 ºC as described above in section 2.3 and was allowed to cool to 25 ºC, where crystal growth was visible as needle crystals. The saturated solution was filtered through a 0.2 µm nylon filter on a Buchner funnel using vacuum. The ibuprofen crystals collected on the filter were scraped off and stored in a tight screw vial for the use in Franz cell experiments.

### 2.7. Membrane treatment

All the membranes in Table 1 were trimmed into circular discs (diameter ~10 mm) sufficient to cover the effective diffusion area of the receptor, and then soaked in receptor fluid for at least 16 hours. Membranes which contained glycerin were rinsed once with receptor fluid before placing them onto the receptor. Membranes such as Cuprophan and polyacrylonitrile AN69 that might crease or fold when wetted, were hydrated as follows: the membranes were sandwiched between two microscope glass slides and submerged in the receptor fluid in a Petri dish. Polycarbonate Nuclepore was applied on the Franz cell with the shiny side facing up, as stated by the manufacturer.

### 2.8. Franz diffusion cell studies

#### 2.8.1. Effect of synthetic membranes on ibuprofen drug diffusion

The ibuprofen drug diffusion from saturated solution across synthetic membranes (listed in Table 1) was investigated using the validated Franz cells and equipment. A clean, dried receptor cell was filled with deaerated 0.1 M sodium hydroxide and allowed to equilibrate at 37 ºC in the heated magnetic block for 15 min. The prehydrated membrane was mounted between the matched donor and receptor compartment and 1 mL of saturated ibuprofen solution was placed on the membrane surface in the donor compartment. All openings including donor top and receptor arm were occluded with parafilm to prevent evaporation. The receptor compartment was stirred at 200 rpm. Using a glass syringe, sample volumes (1–2 mL) were extracted for UV assay (at 272 nm) and fresh preheated replacement medium of same volume was reintroduced into the receptor. Air bubbles formed below the membrane were removed by carefully tilting the Franz cells for the air bubbles to escape via the sampling arm. Intervals between sampling varied from 5 to 30 min. For ibuprofen, crystals were introduced into the donor at approximate hourly interval to maintain its saturated state. The cumulative amount of ibuprofen drug diffusion over 6 h for each membrane was plotted. The ibuprofen drug flux was obtained from the steady state slope of each plot (minimum of 5 replicates). Coefficient of variation (CV) of flux for each membrane was also calculated. (CV = Std. Deviation/Mean ×100%)

#### 2.8.2. Comparison of ibuprofen drug diffusion through membrane of different pore sizes and surface groups

The ibuprofen drug diffusion through cellulose nitrate membranes of two different pore sizes, *i.e.*, 0.1 μm and 0.45 μm were compared. For surface group charges, the ibuprofen drug diffusion through Biodyne B (positively charged) and Biodyne C (negatively charged) were also compared. 

#### 2.8.3. Regression analysis of ibuprofen flux with membrane parameters

The correlations between flux and membrane pore size, MWCO, and thickness (from Table 1) were determined using linear regression analysis. Only membranes with parameters provided by the manufacturers, such as pore size and MWCO, were compared and correlated with flux values. 

### 2.9. Statistical analysis

All statistical analysis was carried out using analysis of variance (ANOVA) and the significance level was accepted when p < 0.05. Post-hoc tukey analysis was also carried out to determine if the means are significantly different from one another.

## 3. Results and Discussion

### 3.1. Drug solubility and mass balance

The average ibuprofen solubility at 25, 33, 37 and 60 ºC was 1.962 ± 0.002, 1.963 ± 0.002, 1.953 ± 0.001 and 2.116 ± 0.08 % w/v, respectively. The ibuprofen solubility measured at these four temperatures was not statistically significant (p = 0.574). The ibuprofen crystal growth was observed as needle-like structure formed in the solution when saturated solution was cooled from 60 to 33 ºC. The pH of ibuprofen saturated solution was 7.2. Typical mass balance results of ibuprofen saturated solution preparations are shown in Table 2. The average of amount of ibuprofen added into the 50 mL of 0.1 M sodium hydroxide for these five preparations was 1.04 ± 0.02; while the coefficient of variation of the mass balance was 1.92%. This typical result showed good repeatability in preparation of saturated solutions.

**Table 2:** A mass balance result (at 60 ºC) for ibuprofen saturated solution.

Preparation #	Amount of ibuprofen added (g)	Amount remaining on the filter paper (g)	Amout of ibuprofen dissolved in 50 mL (g)	Concentration% (w/v)
1	1.657	0.616	1.041	2.082
2	1.621	0.591	1.030	2.060
3	1.637	0.579	1.058	2.116
4	1.620	0.559	1.061	2.122
5	1.767	0.746	1.021	2.042

### 3.2. Ibuprofen drug diffusion across synthetic membranes

**Figure 1:**
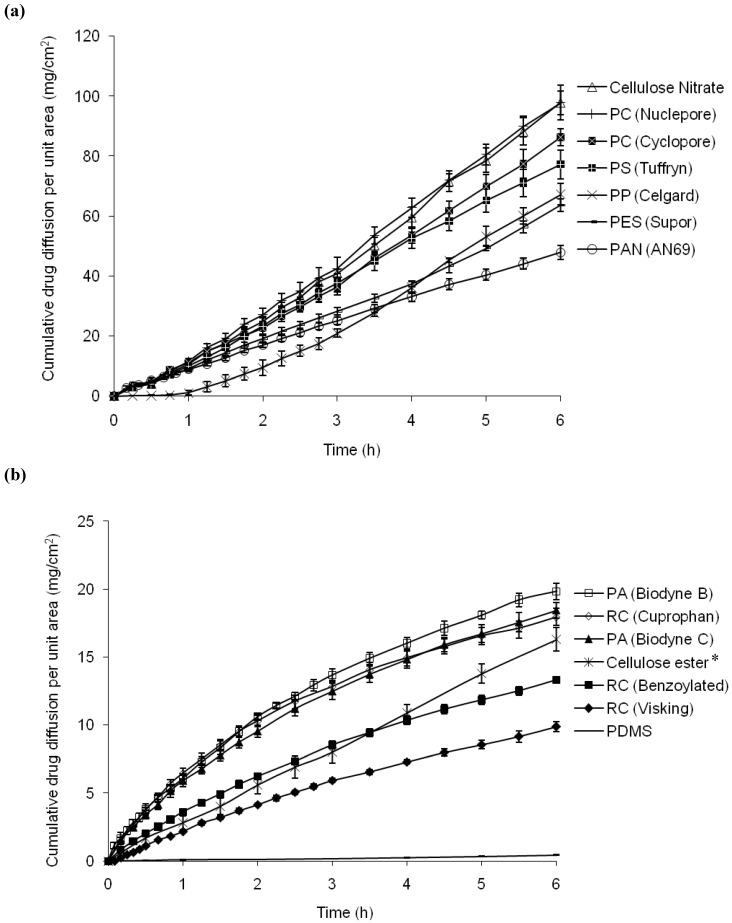
Plots of the cumulative ibuprofen diffusion per unit area over 6 h: (a) high flux membranes (b) low-flux membranes. *membrane may be unstable in the system.

The cumulative ibuprofen drug diffusion per unit area from saturated solutions across the various synthetic membranes over six hours were plotted on two separate scales shown in Figure 1 (a) and (b). The flux values of ibuprofen, total drug diffusion after six hours and coefficient of variation are shown in Table 3. The membranes followed a decreased order of the ibuprofen flux. The ibuprofen flux ranged from the 0.09 (PDMS) to 17.65 (Cellulose nitrate) mg/cm^2^/h. The coefficient of variation (CV) for cellulose nitrate and cellulose esters was above 7% while the rest had CV below 7%. It was observed that the cellulose-type membranes generally produced lower flux while the polymeric-type membranes gave higher ibuprofen drug diffusion.

**Table 3:** Summary of average ibuprofen flux from saturated solution (n = 6), total diffusion after 6 h and the coefficient of variation (CV) of flux for individual synthetic membranes.

Membrane	Flux (mg/cm^2^/h)	Total diffusion after 6 h (mg/cm^2^)	CV (%)
Cellulose nitrate	17.65 ± 2.06	97.89 ± 5.79	11.7
Nuclepore	17.38 ± 0.79	97.75 ± 4.01	4.6
Celgard	15.45 ± 1.02	67.28 ± 3.66	6.6
Cyclopore	14.87 ± 0.50	86.32 ± 2.77	3.4
Tuffryn	13.54 ± 0.49	77.23 ± 4.80	3.6
Supor	10.48 ± 0.31	63.63 ± 2.08	2.9
AN69	8.14 ± 0.38	47.84 ± 2.25	4.7
*Cellulose ester	2.66 ± 0.19	16.30 ± 0.84	7.3
Biodyne B	1.96 ± 0.07	19.32 ± 0.62	3.6
Biodyne C	1.77 ± 0.17	18.44 ± 0.59	4.4
Cuprophan	1.57 ± 0.15	17.94 ± 0.69	4.7
Benzoylated cellulose	1.51 ± 0.04	13.32 ± 0.24	2.9
Visking	1.39 ± 0.09	9.88 ± 0.37	6.2
PDMS	0.09 ± 0.01	0.45 ± 0.002	6.0

* membrane unstable in receptor fluid.

### 3.3. Correlation of flux with membrane parameters

**Figure 2:**
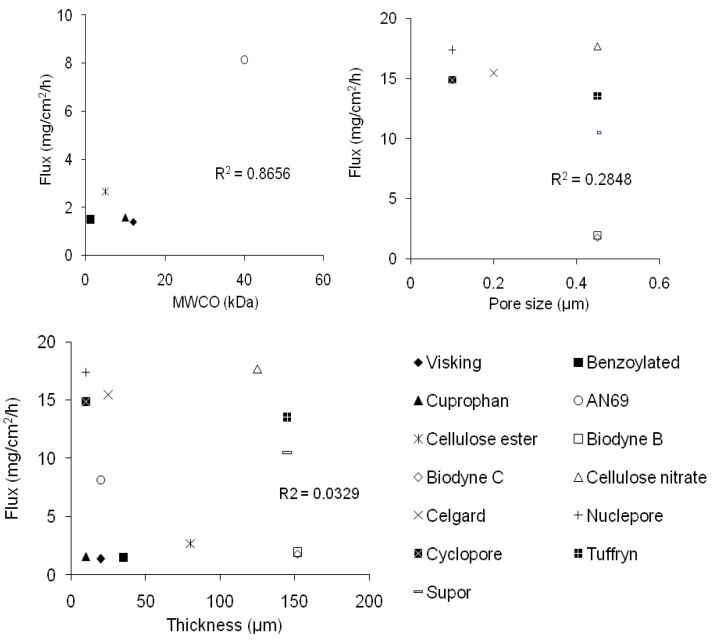
Correlation between ibuprofen flux and membrane molecular weight cut-off (MWCO), pore size and membrane thickness.

No direct strong correlations were found between the ibuprofen flux with membrane pore size, molecular weight cut-off (MWCO), and thickness (R^2^ < 0.99) (Figure 2). 

**Figure 3:**
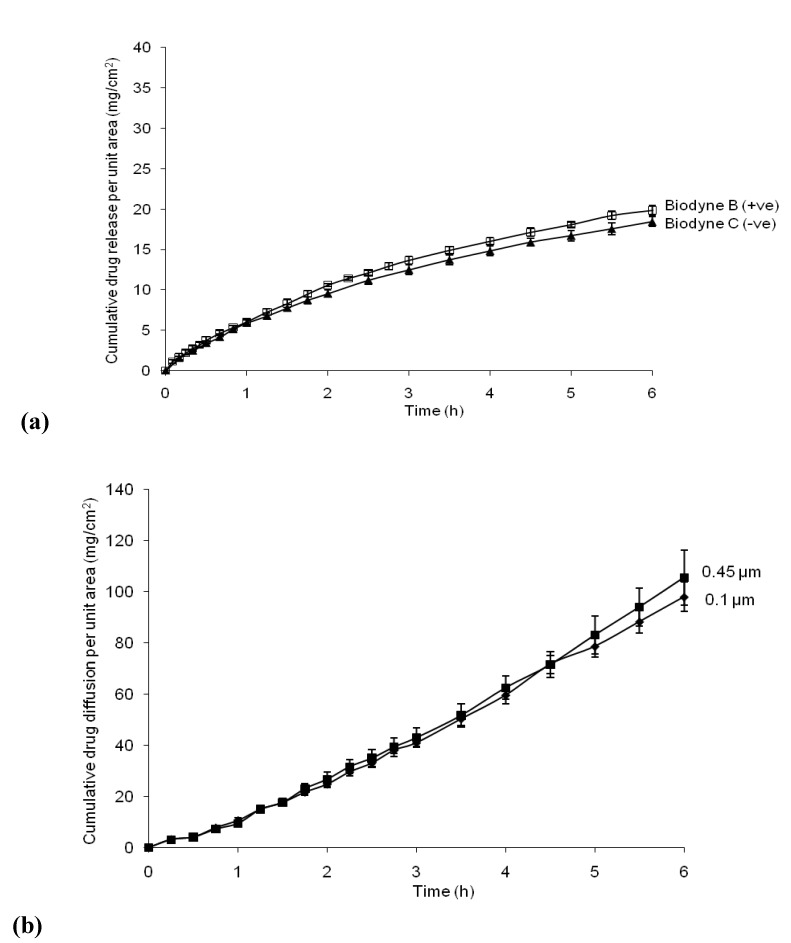
(a) Ibuprofen drug diffusion through cellulose nitrate membrane of different pore sizes (0.45 µm and 0.1 µm) over 6 h; (b) ibuprofen drug diffusion through membrane of different membrane surface groups ( Biodyne B +ve and C -ve) over 6 h.

Pore sizes were correlated within high-flux membranes and MWCO were investigated within low-flux membranes using correlation regression analysis. The cumulative ibuprofen drug diffusion *versus* time plot through membranes of different pore sizes and surface groups are showed in Figure 3 (a) and (b) ,respectively. There was no statistical significance between ibuprofen fluxes over six hours when using membranes of different pore sizes or surface groups. 

### 3.4. Discussion

In this Franz cell experiment, the synthetic membrane was the sole variable factor which affected the ibuprofen drug diffusion, because the apparatus and experimental conditions were maintained constant throughout. The Franz cells and equipment used in this study were validated. The validated parameters include Franz cell dimensions, temperature, membrane treatment, stirring efficiency, as well as sampling frequency. Using the validated Franz cell equipment, the data (CV 2.9–6.6%) obtained in this study suggested that the methodology was robust and reproducible. During the Franz cell run, the saturated state was maintained (with the addition of ibuprofen crystals) throughout the experimentation duration to keep the thermodynamic activity constant and sink condition was maintained via frequent sampling rates (every 15–30 min). Since the experimental conditions were kept constant throughout, the differences in ibuprofen flux values obtained from the different types of membranes were related to the physical properties of the synthetic membranes. Furthermore, the thermodynamic activity was constant; therefore the drug diffusion should be linear. Therefore, any deviation from the linearity should be due to membrane properties. Ibuprofen is a hydrophobic compound (log P = 3.4) and practically insoluble in water (<0.1 %w/v). However ibuprofen was about 20 times more soluble in 0.1 M sodium hydroxide (~2 %w/v). That is also the reason sodium hydroxide was chosen as the receptor fluid in the study because of the high solubility of ibuprofen in sodium hydroxide and hence sink condition can be maintained. The sink conditions were monitored via ‘real time’ ibuprofen calculation, *i.e.,* the ibuprofen concentration was calculated immediately after sampling to ensure the ibuprofen concentration was attained 10% below its saturated solubility that of the donor. From the validation procedure, the temperatures above the membrane were measured as 31.2 ± 0.2 ºC while below the membrane it was 34.1 ± 0.3 ºC. The consistent solubility values indicated that the thermodynamic activity of the drug would not fluctuate greatly within this temperature range. 

In theory, when the porous synthetic membranes are placed in between the donor and receptor in a Franz cell, the pores of the membrane will be filled with receptor medium, hence become the continuation of the receptor medium, while preventing the mixing of donor and receptor content. The results showed that the flux varied across the different porous membranes; some of the synthetic membranes were more rate-limiting to ibuprofen than the others. Different flux values produced indicated that these membranes cannot be simply acting as a physical support. The flux values of the synthetic membranes could be grouped into two plots: 8–18 mg/cm^2^/h (high flux) and 0.1–3 mg/cm^2^/h (low flux). 

It was observed that both high and low flux membranes showed very different ibuprofen drug diffusion profiles. In the former, ibuprofen showed a linear rate of diffusion with time. This drug diffusion profile adhered to the infinite dose zero-order drug release kinetics in which the flux is independent of drug concentration. For the low-flux membranes, the flux profile showed a linear rate of diffusion, but also showed a slight reduction of the gradient after 3.5 h, especially with the Biodyne and regenerated celluloses. 

It is interesting to note that the high-flux membranes were mainly polymeric filter membranes used for microfiltration while the low-flux membranes were cellulose-derived ultrafiltration membranes and nylon membranes. The ibuprofen flux was the lowest for PDMS (non-porous). In this study, four of 10 types of the membranes, namely the polysulfone, the silicone, cellulose esters and polypropylene, were similar to those used in an earlier study [30]. Both studies found that polysulfone membrane (pore size 0.45 μm) was a high-flux membrane. Other than polysulfone, the rest of the membranes from the earlier study did not correspond with the membrane classification in this study. This may be due to drug of a different molecular weight (nitroglycerin) used in the earlier study or because only two replicates were used for each membrane, or both. It was also possible that different fluxes produced were due to the membrane properties such as pore size and thickness. But such information about the membrane was not reported in detail in the earlier study, thus making a comparison between the two studies difficult. 

Correlations studies were carried out to associate the ibuprofen flux with membrane thickness, pore size and MWCO. Nevertheless, no direct linear correlations were established. The ibuprofen flux might be affected by other membrane parameters. Our results also show that the different pore sizes (0.1 *vs*. 0.45 μm) of the same membrane or the same membrane with different surface groups had no effect on the ibuprofen flux.

The underlying theory which describes the transport of drug across a barrier membrane in Franz diffusion cell is the Fick’s Law of passive diffusion, J = K.C_v_ /h, where J is flux, Cv is permeant concentration in vehicle, h is membrane thickness and K is the partition constant of the permeant between the membrane and vehicle. Hatanaka and co-workers attempted to modify Fick’s Law to allow for the fact that drug transport was via the pores, thus the tortuosity and porosity factor of a membrane were included in a modified equation J = D_v_.K'.ε.C_v_ /τ.h [32], where K' is the partition coefficient of the permeant between the solvent in the membrane pore and the vehicle, ε is membrane porosity, τ is tortuosity and D_v_ is the diffusion coefficient of the permeant into the vehicle that fills in the membrane pore. If this equation applies in this study, K', D_v_ and C_v_ were constant; so the ibuprofen flux depended on only three variables, *i.e.,* ε, τ, and h. The microfiltration membrane was known to have a larger pore size (up to 1000 times larger) compared to ultrafiltration membrane. If a microfiltration and an ultrafiltration membrane possessed the same thickness and surface area, the ultrafiltration membrane might have a higher porosity compared to the microfiltration membrane. If porosity is the main determinant of drug flux, the ultrafiltration membranes should have higher drug flux compared to microfiltration membranes. But from this study, most of the ultrafiltration membranes were classified as low-flux membranes, while microfiltration membranes as high-flux membranes. From this observation, tortuosity was thought to have a greater influence in determining drug flux. An example from this study to show that tortuosity was more dominant over porosity was Tuffryn and Supor: Tuffryn and Supor are both polysulfone membranes which possess the same thickness (h = 145 μm) and similar pore sizes (0.45 μm). According to the manufacturer, Tuffryn has lower porosity (ε ~ 60%) compared to Supor (ε ~ 80%). However, the ibuprofen fluxes across Tuffryn and Supor were the reverse of the membrane porosity values, with Tuffryn 13.54 ± 0.49 mg/cm^2^/h and Supor 10.48 ± 0.31 mg/cm^2^/h, respectively. So membrane tortuosity (values not provided by the manufacturer) was thought to be the factor determining the ibuprofen flux. And from this study, it was deduced that the Supor membrane was more tortuous compared to Tuffryn. 

However, when both membranes possess similar tortuosity, porosity will play a more important role in determining drug flux [32]. This phenomenon was observed with the polycarbonate Nuclepore and Cyclopore membranes. Both of the polycarbonate membranes were manufactured by the Track-etched process where the pores generated are cylindrical in shape, transversing across the membrane (τ~1). However, the observation that Cyclopore had lower ibuprofen flux (14.87 ± 0.50 mg/cm^2^/h) than Nuclepore (17.38 ± 0.79 and mg/cm^2^/h) might be due to the slightly lower membrane porosity of Cyclopore (4%) compared to Nuclepore (8%). 

Additional membrane support is incorporated to improve membrane integrity, but this may affect the transport across the membrane. Tuffryn and Biodyne membranes possess the same nominal pore sizes (0.45 µm) and relatively similar thickness (145–152 µm). Despite the similar physical characteristics, the Tuffryn was categorized as a high-flux membranes while Biodyne was a low-flux membrane. Tuffryn is a self-supported membrane, *i.e.* the membrane does not contain any additional layer or support. Biodyne membrane contains non-woven polyester support (thickness 60–90 μm; finished membrane thickness 152 ± 13 μm). This support may be the additional tortuous path for drug transport.

The presence of membrane coating served as a protective layer for the membrane. This coating may be barrier or hindrance to drug diffusion. Celgard possesses a proprietary coating, but specific chemical identity of the coating is protected by the manufacturer as a trade secret (Hoechst). In this study, lag time (~1 h) was observed when Celgard was employed. It was deduced that the lag time may be associated with this coating. Celgard was not suitable for Franz cell studies because the lag time essentially masked vehicle performance, especially during the first hour of the experiment. Care should be taken when selecting a porous membrane for Franz cell experiments, preferentially using one without any coating or any additional physical layer.

AN69 is an improved hemodialysis membrane and is well known for its high permeability because it is very thin compared to the conventional cellulosic dialysis membranes [33]. AN 69 has been categorized as a high-flux membrane in the hemofiltration context [34]. The same trend was seen in this Franz cell study, in which the AN69 produced higher ibuprofen flux compared to the cellulose membranes. As a result, it was grouped as a high-flux membrane in Franz cell drug diffusion investigation. 

The regenerated cellulose (Visking, Cuprophan, Benzoylated cellulose) and cellulose acetate were classified as low-flux membranes. Cellulose nitrate was an exceptional cellulose derivative which was high-flux. Unlike the other cellulose-modified membranes, ibuprofen drug flux across the cellulose nitrate was very rapid (17.65 ± 2.06 mg/cm^2^/h) and not significantly different from Nuclepore. Assuming all cellulose-type membranes possessed similar tortuosity, the rapid ibuprofen flux across the membranes was due to the high porosity of cellulose nitrate membrane (66–84%). 

Among the low-flux cellulose membranes, cellulose acetate produced the highest ibuprofen flux. It was found that the cellulose acetate used in this study was not compatible with the receptor fluid. According to the manufacturer, the membrane was only stable in pH 3–8 while the receptor fluid (0.1 M sodium hydroxide) had a pH of 12. It was likely that the membrane integrity had been weakened and ibuprofen transport across the membrane became less resistant. This may also explain why such a high CV (7.3%) was obtained. Consequently, the investigator should always be very cautious when choosing a membrane which is compatible with the receptor media especially if the experimental period is very long. The membrane must also be able to withstand long exposure to chemical solvent.

Although Visking, Cuprophan and Benzoylated are regenerated celluloses, the ibuprofen fluxes generated were different. Among the three cellulose membranes, Cuprophan gave the highest flux, followed by Benzoylated tubing then Visking. Cuprophan is an ultrathin cellulose membrane produced via cuproammonium process; ibuprofen passed quickest through Cuprophan. Ibuprofen diffused through the Benzoylated cellulose at a faster rate compared to Visking. This was because Benzoylated is relatively hydrophobic compared to Visking due to the presence of benzoyl groups on the surface. 

PDMS is an isotropic polymer widely used as an alternative model barrier for prediction of drug permeation across skin [35]. The drug diffusion across PDMS follows Fick’s law and possesses hydrophobic properties like skin, so making it a good model for the stratum corneum. Here, the PDMS produced the lowest ibuprofen flux. This was because the PDMS matrix was limiting the drug diffusion process. This indicates that PDMS was not suitable for formulation investigation because it can limit the drug permeation and thus mask the formulation vehicle effect. In contrast, cellulose nitrates and Nuclepore were found to be the least rate limiting for ibuprofen so they are both suitable for screening of formulation which contained ibuprofen. 

## 4. Conclusions

Porous membranes derived from various polymers demonstrated different degrees of diffusional resistance to ibuprofen. This indicates that there would be wide discrepancy in results obtained from different laboratories using different porous synthetic membranes. This would also incur disparity in drug diffusion profiles in product quality control, which may not be the result of the formulation itself. Based on the ibuprofen drug diffusion tests, the synthetic membranes are categorized into high and low flux membranes according to the rate of ibuprofen drug diffusion over six hours. The high flux membranes are typically microporous membranes whereas the low flux membranes consist of ultrafiltration membranes. The following factors should be considered when selecting a porous synthetic membrane for quality control: 1) The ideal high flux membrane for formulation analysis should have high porosity (> 60%), tortuosity of 1, and be relatively thin (~10 μm), 2) Synthetic membranes for microfiltrations are preferred for Franz cells studies compared to membranes for other applications, 3) Membranes with coatings were not favorable, 4) If the membrane contains filler support, the investigator must be cautioned that the filler support did not have an effect on drug flux, 5) Other factors to be considered are the compatibility of the membrane with the donor and the receptor component as well as the cost effectiveness of the membranes. 

Although there are various types of commercial ‘porous’ membranes available in the market, each type may produce different drug diffusion properties. In general, investigators must exercise caution in choosing the most appropriate membrane to be used with Franz cells for topical quality control assessment.
